# Music Undergraduates' Usefulness and Importance Expectations: The Bologna Process from an Australian University Perspective

**DOI:** 10.3389/fpsyg.2016.01054

**Published:** 2016-07-12

**Authors:** Dominic G. Harvey, Jane W. Davidson, Chenicheri S. Nair

**Affiliations:** ^1^School of Music, The University of Western AustraliaPerth, WA, Australia; ^2^Melbourne Conservatorium of Music, The University of MelbourneMelbourne, VIC, Australia; ^3^Office of the Deputy Vice-Chancellor (Education), The University of Western AustraliaPerth, WA Australia

**Keywords:** Australia, Bologna Process, music undergraduates, expectations, usefulness, importance

## Abstract

The Bologna Process model of higher education has been introduced into some Australian universities since 2008. This model promoted university study through a liberal arts philosophy that advanced a worldview approach at the undergraduate level. The model generalized the student experience and eliminated undergraduate specialization. An interesting situation for music undergraduate study thus arose. Expertise and expert performance research has argued an opposing educational approach, namely: Extensive long-term commitment through focused practical engagement and specialized tuition as prerequisites to achieving musical mastery, especially in performance. Motivation research has shown that the majority of this specialized development in pre-university years would be accessed and reinforced predominantly through private music tuition. Drawing on this contextual literature, commencing university music undergraduates would have expectations of their prospective study founded from two historical influences. The first: How undergraduates had accessed pre-university music tuition. The second: How and in what ways undergraduates' pre-university musical activities were experienced and reinforced. Using usefulness and importance measures, the study observed the expectations of students about to commence music undergraduate studies at three representative Australian university music schools. One of these universities operated the Bologna styled model. No other known Australian study has investigated this implementation for any effects upon music undergraduate expectations. How much commencing music undergraduates would draw on their pre-university music instruction and experiences to predict their usefulness and importance expectations formed the basis for this investigation. Strong relationships between usefulness and importance were found across all units of study. Despite strong correlations across all units of study between usefulness and importance, there was a reluctance to be outwardly positive toward units of study that were not practical and performance-related, such as Music History. The educational model did not appear to affect music undergraduate expectations.

## Introduction

This paper reports data that were collected in 2013, which came from within a three-year longitudinal research project (2012–2014), and that tracked music undergraduates from commencement to completion of their degree programs. The research followed a mixed methodological design underpinned by a pragmatic, worldview paradigm (e.g., Onwuegbuzie et al., 2009; Creswell, 2010, 2014; Tashakkori and Teddlie, 2010). A previous paper (see Harvey, 2014) reported commencing music undergraduate expectation data from one university music school in 2012. That observation took place from within the university that had introduced the Bologna Process model. Perceptions data came from the first ever music cohort to experience that new undergraduate degree program. The present study investigated expectation perceptions of commencing music undergraduates at three representative Australian university music schools in 2013. Two of these universities operated a liberal arts and the other a conservatorium styled music undergraduate degree program. The current study investigated the extent to which commencing music undergraduates relied upon pre-university music instruction and experiences to inform usefulness and importance expectations of a liberal arts-styled music undergraduate degree program. To help understand this research question, the investigation was guided by two angles of inquiry:

- The expectation perceptions of imminent university music study for commencing music undergraduates and,
- Any effect of the implementation of the Bologna Process at The University of Western Australia (UWA).

As a mixed methodological design, and in conjunction with the nature of its paradigm, the study observed differences and similarities in the expectations of commencing music undergraduates across the three universities. This exploratory platform served to suggest interpretations of student expectations rather than to test hypotheses as in a scientific experiment and or through using a control. Thus, the following review contextualizes the issue from the established research literature that has underpinned this investigation.

Research has demonstrated that achieving superior music performance proficiency has required long-term commitment to frequent participation and active engagement in performing, composing, and or studying music (e.g., Howe et al., 1998; Hunt, 2006; McPherson, 2006). Firstly, sustained, systematic and regular deliberate practice regimes must be devised, implemented and routinely repeated and updated (e.g., Ericsson et al., 1993; Amirault and Branson, 2006; Lehmann and Gruber, 2006). Secondly, receiving expert and specialized music instruction (usually one-to-one) was a critical element (e.g., Amirault and Branson, 2006; Lehmann and Gruber, 2006). Finally, it was most preferred and considered necessary for the developmental process to commence from as early an age as possible. A number of notable musicians have demonstrated this (e.g., The Beatles, Mozart, Bach, Haydn, Beethoven; in Weisberg, 2006, p. 770). The benchmark for achieving mastery in music performance has been established to be around 10,000 h, otherwise referred to as “the 10-year rule” (Ericsson, 2006, p. 685; Kellogg, 2006, p. 398; Simonton, 2006, p. 327). As a backdrop to the present study, it was assumed that most students about to commence music undergraduate study had achieved a daily deliberate practice commitment commensurate with or close to that benchmark to be considered “good experts.” Beyond this, only certain students would have achieved the requisite practice hours to be considered “best experts” (Ericsson et al., 1993). Whilst not the focus of the current study, practice commitment could not be ignored, as it formed a critical foundation to the main issues investigated. For example, those students who were used to higher levels of daily practice prior to university would have different expectations toward a liberal arts-styled music undergraduate study program than those who were not. Differing expectations may also be related to the type of instrument taken up before university that would demand varying degrees of application to practice (Lehmann and Gruber, 2006). Additionally, the type of music instruction and music performance activities (e.g., solo and ensemble) accessed prior to university would influence usefulness and importance expectations. This issue is explored next.

In relation to pre-university specialized music instruction access, research by Amirault and Branson (2006) and others has concluded that the predominant entry point of specialization would be through the independent private music studio/teacher rather than school classroom music. Students of music might experience both of these points of access prior to university. For example, some Australian schools (state, independent and private) run in-school music programs, while others do not and have had to rely more upon accessing music tuition privately (e.g., Davidson and Burland, 2006; McPherson and O'Neill, 2010). It would be logical to conclude that point of access to music is influential in motivating students to pursue further study at university. Related to this, whether the music instruction and related activities were solitary or ensemble is also significant. Student expectations when commencing university may be influenced by previous solo and ensemble music instruction and activities. For example, Ericsson et al. (1993) found that what separated the “best” violinists from all others was the amount of daily solitary (deliberate) practice that both teachers and students expected. These “better” violinists would have more emphasis placed upon solo instruction and performance, and therefore greater expectations for this to continue, than those who did not. The present study anticipated that: The majority of commencing undergraduates would have accessed their pre-university music tuition privately; the majority of pre-university music instruction and activity would have had a solo focus. Irrespective of the focus (solo or ensemble), music performance would have been the primary pre-university activity. This matter is now addressed.

Together, the type of music instruction encountered and the focus of practical music activity prior to university would likely have shaped the expectations of students (Lehmann and Gruber, 2006). For example, for some, an instructional emphasis upon and active application to solo performance (and subsequent demands for solitary deliberate practice) would be expected to continue into university. Conversely, a student from pre-university music training that promoted high levels of ensemble participation—such as through classroom music—might expect a more inclusive, participative approach to continue into university. Such a student may also have lesser expectation for high levels of solitary deliberate practice. Additionally, students whose pre-university focus in music instruction and activity lay in areas outside of specialized performance such as music theory and composition might have different expectations toward university study. Finally, commencing undergraduates that were undecided about their specific focus in music may have come to university anticipating that a specialization would emerge over time. Interpreting Bandura's (1977a,b) efficacy expectations: Previous experiences, role modeling, socio-cultural environments, and the psychophysiological reinforcements encountered by, in this case, music students in pre-university music training would have contributed to defining individual preferences toward particular units of music study about to be encountered at university. The present study anticipated that: A spectrum of commencing music undergraduates would be encountered; the major focus of pre-university music instruction and activity would be upon performance.

As has been proposed, discriminating preferences toward specific future music specialization would have been reinforced through pre-university music instruction and related music activities (Bandura, 1977b; Hunt, 2006). Pre-university influences would shape undergraduate preferences and choices based upon, for example, expectancy-value, particularly in relation to music performance (e.g., Fishbein and Ajzen, 1975; Ajzen, 1991). In other words, the predominantly performance based pre-university instruction would have influenced commencing music undergraduates to value performance based units of university study more highly than those that were purely theoretical/academic. From representative literature on expertise development and achieving success (e.g., Ericsson et al., 1993; Amirault and Branson, 2006; Dweck, 2006; Feltovich et al., 2006), a further consideration for this study had to be taken into account. That is, the extent to which music performance activities focused upon solo or ensemble outcomes would influence the orientation of subjective value expectations in relation to utility. Not only would performance based units of study be more valued than those that were theoretically/academically grounded, they would have greater utility to achieving a particular outcome. The expectancy-value theory of achievement motivation research (e.g., Wigfield and Eccles, 2000; Eccles and Wigfield, 2002) served also to underpin the current investigation. Thus, measures of usefulness and importance were used to help gauge commencing undergraduate expectations for the particular units of music study about to be encountered at university for the first time.

The rationale for undertaking this study was motivated by the global changes that have taken place in higher education since 2000. Historically, music undergraduate education in Australia was accessed through the music conservatorium, which emphasized the master–apprentice (one-to-one) methodology of practical instruction (Bennett, 2008a). Outcomes focused almost exclusively on the development of the music performer (Walker, 1990; GAP, 2011). Following federal higher education reviews during the 1980s and 1990s (e.g., Dawkins, 1988), music conservatoria were largely absorbed into research-intensive universities. The Bradley review (Bradley et al., 2008) increased pressure for universities to perform to international research standards (Australian Government, 2012). The environment of the music “hot-house” that had nurtured small numbers of domestic specialist music students in Bachelor of Music (BMus) programs was effectively extinguished (Bennett, 2008a,b; Stowasser, 2008). With the Bologna Declarations of 1988 and 1999 fundamental changes were made to the way higher education would be structured and delivered in Europe (Reinalda and Kulesza, 2006). The disestablishment of the traditional operations of university “long degree” undergraduate specializations from 2000 (Assefa and Sedgwick, 2004) and transformation into the European Higher Education Area by 2010 became known officially as the Bologna Process (Reinalda and Kulesza, 2006).

Since 2000, the 3-year model of a general “worldview” undergraduate education approach has had international influence through adoption of the Bologna Process by universities in North America and Australia (e.g., Woessmann, 2011; Ralston et al., 2011). Philosophically, the Bologna Process model provided access for all to undergraduate education. It has been argued that with a 3-year undergraduate degree, a broadly educated and skilled population would be ready either to enter the workforce or undertake further specialized postgraduate education (Assefa and Sedgwick, 2004). Based upon this background information, it was considered appropriate for the current study to compare two liberal arts-styled models with a conservatorium-styled model of music undergraduate education that operated in representative Australian university music schools. This comparison would help to understand any differences between the expectations of commencing music undergraduates across the universities. The University of Western Australia (UWA) introduced its version of the Bologna Process in 2012 under *The New Courses Framework* (The University of Western Australia (UWA), 2008). This saw the disestablishment of some 170 individual degree specializations into five generic degree programs (ibid.). For example, the Bachelor of Music degree (BMus) became the Bachelor of Arts (BA) within which Music Studies Majors could be taken. Other Australian institutions such as The University of Melbourne, wherein lay the Melbourne Conservatorium of Music (MCM), despite its name introduced a similar undergraduate degree model in 2008. For Melbourne, the model was adopted from the American “Ivy League” liberal arts undergraduate framework (Assefa and Sedgwick, 2004). The Western Australian Academy of Performing Arts (WAAPA) within the Edith Cowan University (ECU) was an institution that had retained its conservatory-styled degree program in music (Edith Cowan University (ECU), 2013). In all cases, the music undergraduate degree programs under the Australian tertiary education framework comprised of six 13-week semesters.

From this context the research question was proposed: How much would commencing music undergraduates rely upon pre-university music performance instruction and experiences to inform their expectations of a liberal arts styled university degree program? There were two assumptions that guided the first part of the research question. Firstly, that pre-university music performance instruction and activities would positively affect undergraduate expectations of university study. These expectations would be positive irrespective of the pre-university instructional access, methodology, emphasis, or specialization. Secondly, music undergraduate expectations would reflect specific subjective value preferences toward particular areas of study. These preferences would reflect perceptions of greater utility in relation to particular outcomes. This would mean that practical units of study such as Music Performance would be considered more useful and important in the immediate and longer terms than academic units of study such as Music History. In short, music undergraduates' subjective value preferences based upon their pre-university experiences of music instruction and activity would positively influence their expectations of university study. To answer the second part of the research question, these assumptions of positive participant responses were made despite the potential effect a non-specialized undergraduate degree program model might have upon individual perceptions of utility, and whether or not the institution of study provided a liberal arts or conservatorium styled music undergraduate degree program.

## Materials and methods

### Participating music institutions

In 2013, three institutions participated in this study; the School of Music (SoM) at The University of Western Australia (UWA), the Melbourne Conservatorium of Music (MCM) within The University of Melbourne (UM), and Edith Cowan University (ECU) music school situated within the Western Australian Academy of Performing Arts (WAAPA). As introduced previously, there were two angles of inquiry taken to address the research question, which were to investigate:

- The expectation perceptions of imminent university music study for commencing music undergraduates and,
- Any effect of the implementation of the Bologna Process at UWA.

Questioning of participants in the study was made unilaterally consistent without distinguishing between the institutional models. This was to eliminate bias analysis.

### Participants

Commencing music undergraduates were invited to participate in the study and participant expectations were observed immediately prior to the commencement of the first semester of study. The UWA SoM (*N* = 34), ECU (*N* = 65). and MCM (*N* = 108) cohorts were provided consent forms and an information sheet detailing the study and its ethical status at the first all-school meeting held at the respective institutions at the start of the academic year. Participants were advised through the information sheet that involvement in the study was completely voluntary and that withdrawal could be made at any time and without having to state a reason. The information sheet can be referred to in the Appendix (Supplementary Material).

### Questionnaire

Questionnaire items provided to research participants had both quantitative and qualitative information. Likert scale items (1 = “not at all useful/important,” 2 = “somewhat useful/important,” 3 = “useful/important,” 4 = “very useful/important,” 5 = “unit not taken,” and 6 = “not applicable”) provided quantitative data for student expectations of university music units of study. Because of the differences between institutions regarding the nomenclature of individual units of study, each unit of study in the respective curriculum was accommodated into the questionnaires for each cohort of UWA, MCM, and ECU. Scale points 5 and 6 were discarded from the reporting, as they did not provide any meaningful information for the current study. The units of university music study from each institution have been presented under data preparation below, as they required categorization in order to facilitate analysis.

Qualitative data were collected from the following partially open-ended items: How pre-university music instruction was accessed (e.g., school/private); the type of pre-university music experience emphasis (e.g., practical/academic); and the focus of pre-university music instruction and performance (e.g., solo/ensemble). Choice options provided in the questionnaire for these items were informed through literature and industry experience. The questionnaire items reported in this paper are found in the Appendix (Supplementary Material).

### Data preparation

As explained in the introduction to this paper, ECU had retained its conservatorium-styled BMus program in comparison with UWA and MCM. The individual units of music study at ECU were directly related to specific musical studies in respect to what they offered undergraduates. The UWA and MCM units of study reflected the liberal arts degree structure. Through communications with the institutions, the researchers were able to identify which of the units of study were practical based (i.e., performance) and which were academic (i.e., history). The worldview unit (Broadening for UWA and Breadth for MCM) applied only to those institutions. For data analysis in this study, units of music study were thus categorized generically as: Music Performance, Music History, Music Aural and Music Ensemble. For UWA and MCM participants, the worldview unit (Broadening and Breadth respectively) was categorized as General. The breakdowns of each institution's curriculum structure for the music degree program have been presented in Tables 1, 2.

**Table 1:** **Bachelor of arts: UWA music studies/specialist music studies**.

Year 1	Semester 1	MUSC1310 communication skills in music	MUSC1321 music language 1	MUSC1341 practical music 1	Broadening unit

Source: UWA Handbook (The University of Western Australia (UWA), 2013).

**Table 2:** **Bachelor of arts: UWA music studies and second major**.

Year 1	Semester 1	MUSC1310 communication skills in music	MUSC1321 music language 1	MUSC1341 practical music 1	Second major

Source: UWA Handbook (The University of Western Australia (UWA), 2013).

Tables 1, 2 show the Year 1 Semester 1 music units for UWA SoM commencing music undergraduates, 2013. Table 1 shows the structure of the BA with Music Studies Major and Specialist Music Studies sub-Major. Table 2 shows the same information but for the Music Studies Major with Second Major. The Second Major was the Music Ensemble unit.

Table 3 shows the Year 1 Semester 1 music units for ECU commencing music undergraduates, 2013, and shows the structure of the BMus with the Performance Major. ECU did not have the General unit within its curriculum structure.

**Table 3:** **Bachelor of music: ECU music studies (performance)**.

Year 1	Semester 1	MUS1116 principal studies 1	MUS1104 ensemble and extension studies 1	MUS1106 music techniques 1	MUS1111 music history and culture

Source: ECU Handbook (Edith Cowan University (ECU), 2013).

Table 4 shows the Year 1 Semester 1 music units for MCM commencing music undergraduates, 2013. Table 4 shows the structure of the BMus with the Performance Major. The Breadth Unit was the General unit not related to music.

**Table 4:** **Bachelor of music: MCM music studies (performance)**.

Year 1	MUSI10026 music performance 1	MUSI10025 writing about music	MUSI10204 aural studies 1	Applied skills unit	Breadth units

Source: UM Handbook (The University of Melbourne (UM), 2013).

### Quantitative data analyses

As exploratory mixed methods research, the present study and its larger longitudinal project have analyzed and reported observational information. The utilization of control and experimental groups and certain tests of the data within a strictly scientific methodology were not considered applicable to this research. This was because the interpretation of results could only achieve suggestion, rather than generalization across populations. For these reasons, tests such as Mann-Whitney and Kruskal–Wallis were not selected, as the study did not use a control group. Rather, analyses concentrated upon identifying potential relationships between perceptions of usefulness and importance of university music units. These tests were then compared across the participant groups to establish any similarities or differences. Given the comparatively small sample sizes, the researchers selected correlational tests using the Spearman's rho coefficient. The four-point Likert scale similarly was regarded as sufficient given the parameters of the research. Although it has been identified as a controversial issue (e.g., Hills, 2008), the Likert scale data used in this study were treated as ordinal scales insofar as they related to usefulness and importance preferences. This in itself was seen to preclude Kruskal–Wallis tests.

### Qualitative data analyses

The qualitative data could be treated quantitatively as these questionnaire items contained static choice options that covered the most likely response possibilities. The “other (please describe)” option allowed for any potential choice that had been omitted in the list provided in the questionnaire item. This meant that the results would show descriptive tendencies rather than statistical significances and or levels of agreement and disagreement amongst the cohorts. These data were considered to be an important inclusion in order to frame the assumptions that music undergraduates would draw upon previous music instruction and experiences to inform their predictions of university study. As nominal data, the qualitative inputs were helpful to shed light upon: The types of students the respective music institutions had attracted; the commitment students had made to practice and study prior to university commencement; the type of instruction and experiences music undergraduates had experienced before university and would likely continue to expect at commencement of the first academic year.

## Results

**Figure 1:**
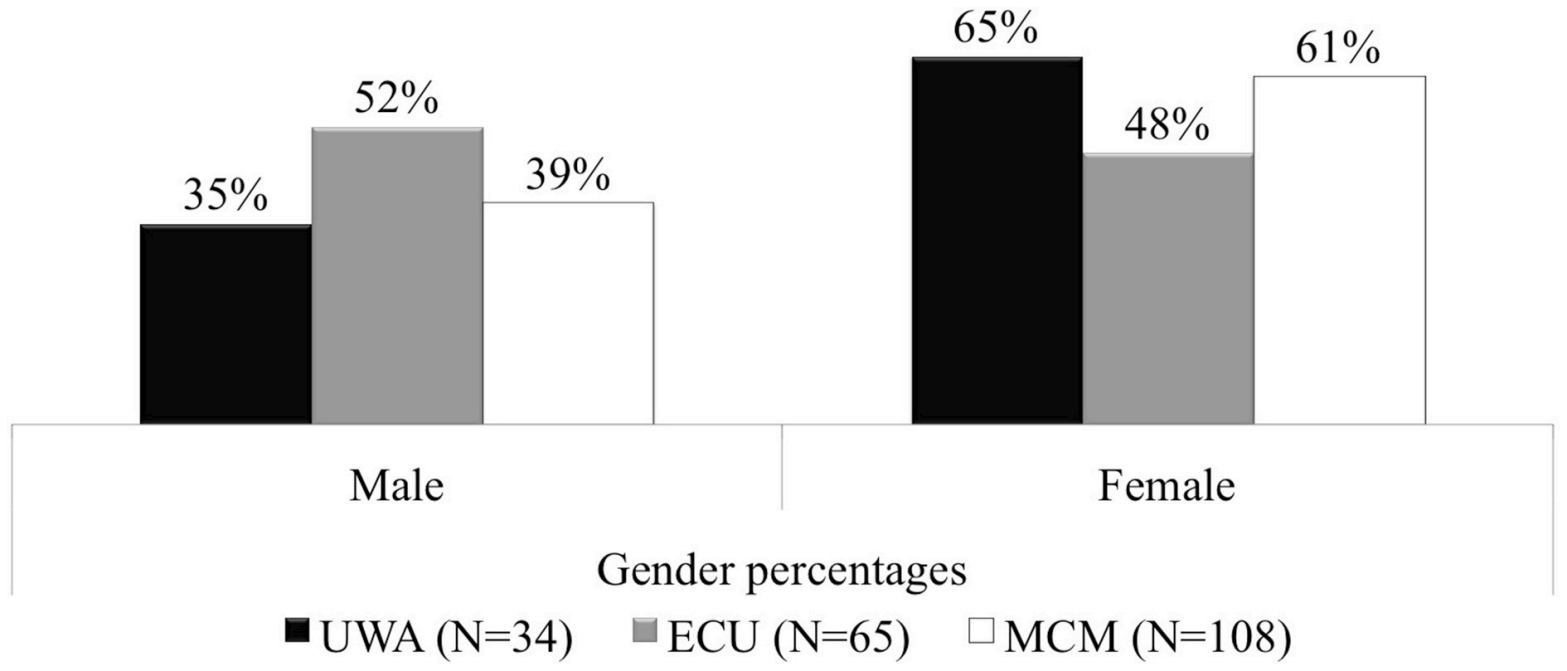
**Gender percentage distributions: UWA, ECU, and MCM Semester 1, 2013**.

Figure 1 summarizes the demographic breakdown of music undergraduates at the universities. At the UWA and MCM female commencing undergraduates (65 and 61% respectively) outnumbered males (35 and 39% respectively). ECU commencing male participants (52%) slightly outnumbered females (48%).

Table 5 reports the various music instrument families—for example, strings as opposed to violins or brass as opposed to trumpets—at the commencement of first semester 2013. Commonly across the universities, higher proportions of piano/keyboard and orchestral woodwind instrument families were observed. That is from 18% (UWA) to 22% (MCM) for piano/keyboard and from 14% (MCM) to 20% (UWA) for orchestral woodwind. Results suggested that respective university music schools exhibited particular strengths for pre-university music students seeking access to tuition in instrumental groups at the undergraduate entry level. For example, the UWA attracted 20% of its commencing cohort as string players, whereas the ECU was just 1%. Where Jazz formed part of the choice for commencing music undergraduates, the ECU and MCM had attracted 3 and 5%, respectively of band woodwind instruments, while UWA had none presumably because it did not offer Jazz.

**Table 5:** **Percentage instrument distributions: UWA, ECU, and MCM Semester 1, 2013**.

	**pf/kbd (%)**	**Perc (%)**	**v (%)**	**Orch ww (%)**	**br (%)**	**str (%)**	**bd ww (%)**	**gtr (%)**	**Other (%)**	**Comp (%)**
UWA *N* = 34	18	9	9	20	15	20	0	6	3	0
ECU *N* = 65	20	11	32	17	8	1	3	8	0	0
MCM *n* = 78	22	1	27	14	11	13	5	3	4	0

The instrumental category abbreviations in this table follow convention according to http://imslp.org/wiki/IMSLP:Abbreviations_for_Instruments. From left to right these were: Piano/keyboard, percussion, voice, orchestral woodwind, brass, strings, band woodwind, guitar, other specialized instrument, and composition.

Commencing voice student percentages showed strengths of tuition in this area for ECU (32%) and MCM (27%). The result for UWA (9%) reflected downsizing changes in vocal staffing at UWA that occurred in 2013. The Composition category had no subscription across the three universities. This result did not imply that there were no undergraduates majoring in composition, rather, the questionnaire item had requested information concerning the instrument that commencing undergraduates played. Some participants may have declared their primary musical instrument while intending to study composition as a major. Alternatively, commencing music undergraduate instrumentalists would have the option to change study preference to composition, such as was allowed at the commencement of the second year of study under UWA's degree structure. The results reported in Table 5 provide a context as to the type and proportion of music undergraduates the study had observed.

### Expertise and expert performance—pre-university daily practice/weekly study

**Figure 2:**
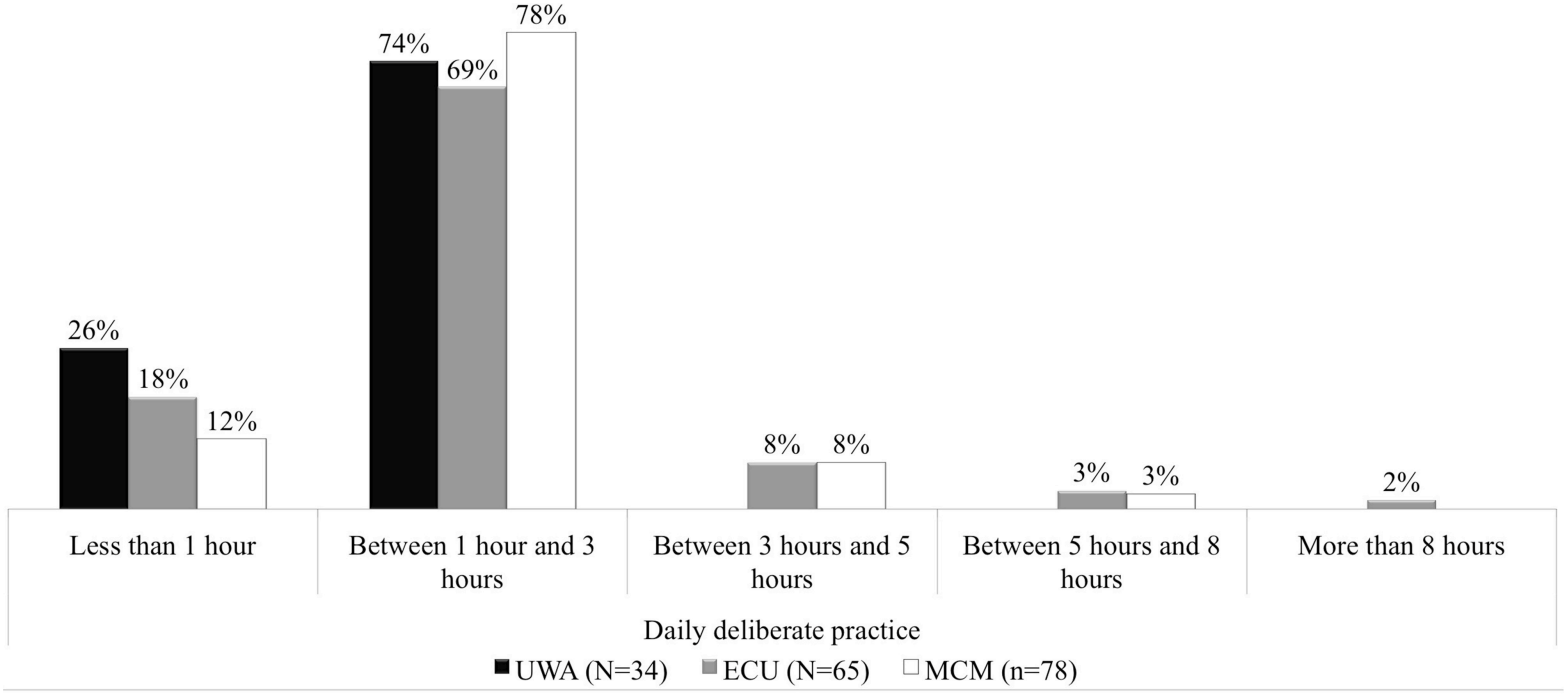
**Percentage daily practice commitment: UWA, ECU, and MCM Semester 1, 2013**.

Results in Figure 2 show the estimated pre-university commitment to daily deliberate practice. Seventy four percent (74%) of commencing music undergraduates across the schools estimated between 1 and 3 h, with 19% applying < 1 h per day in practice. ECU results had the only comprehensive representation across the five time categories provided, with 2% (*N* = 65) having committed themselves to more than 8 h per day to practice. On the other hand, UWA results were split across the first two categories only. More of the undergraduate contingent from MCM (78%) fell within the second time category (1–3 h).

**Figure 3:**
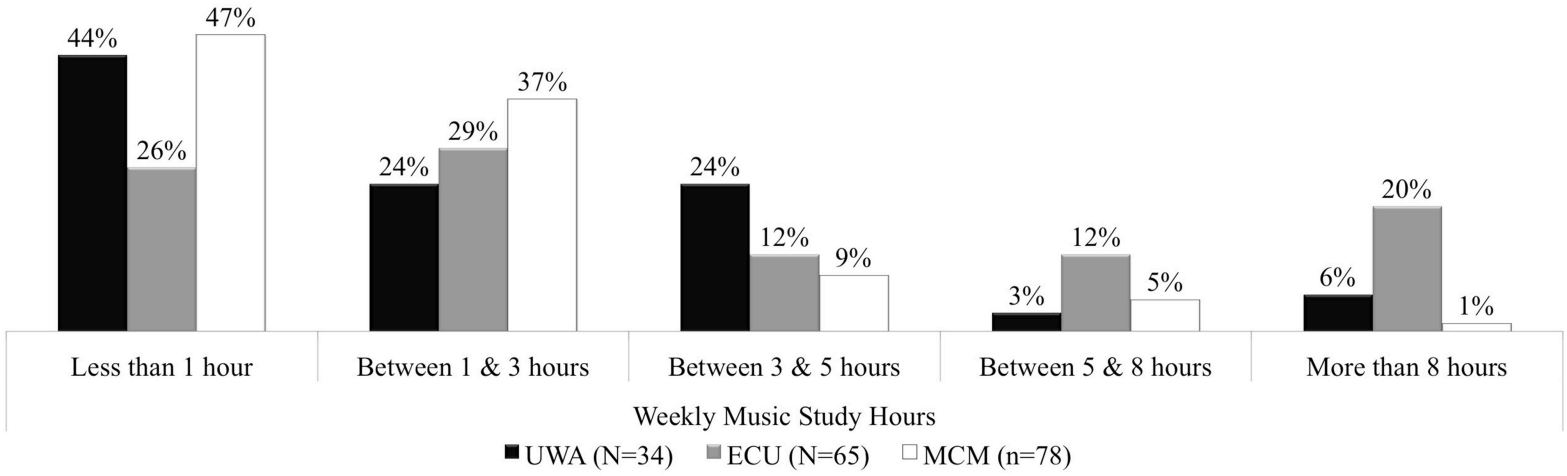
**Percentage weekly study commitment: UWA, ECU, and MCM Semester 1, 2013**.

Figure 3 reported percentages for pre-university weekly commitment to the academic study of music (such as reading about music, writing about it, music history, composing, harmony training). More students from UWA (44%) and MCM (47%) did less than 1 h per week than did ECU students (26%). More students at MCM (37%) and ECU (29%) committed between 1 and 3 h than UWA (24%). However, students at UWA applied themselves to between 3 and 5 h (24%) more so than ECU (12%) and MCM (9%). ECU results showed greater distribution across the time allocation categories, with 12% committing to between 5 and 8 h and 20% to 8 h or more per week.

### Pre-university music instruction and activity—access

**Figure 4:**
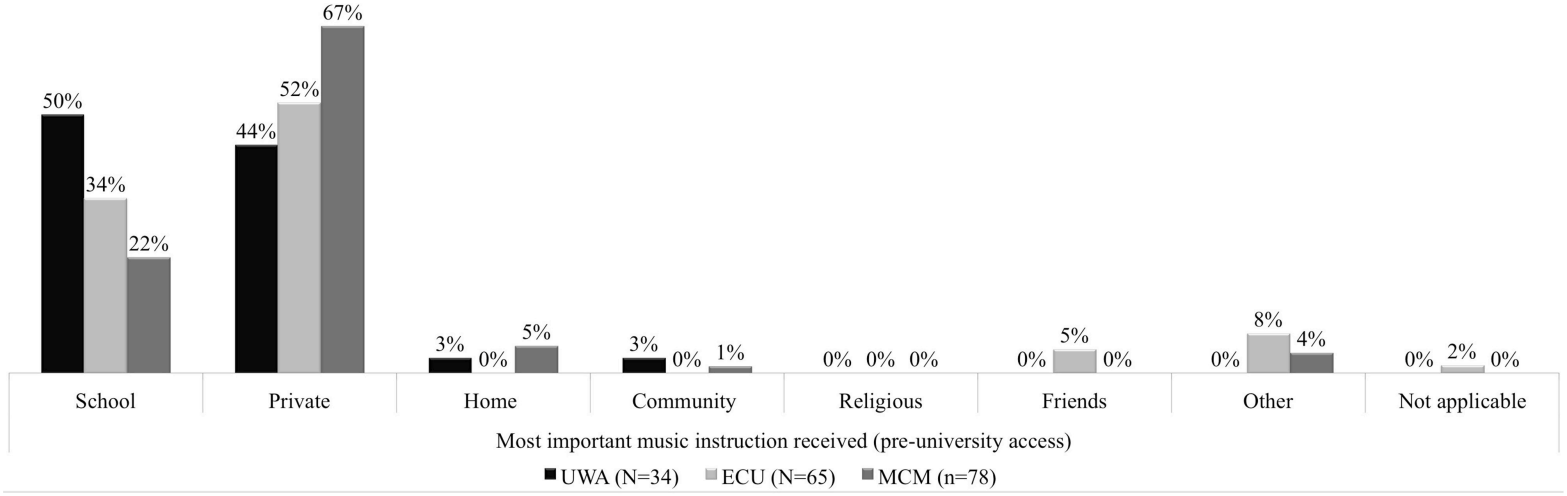
**Percentage most important pre-university instruction received—access**.

Combined averages for UWA, ECU, and MCM (*n* = 177) in Figure 4 illustrated that in 2013 54% of students accessed their most important pre-university music tuition privately, and 35% did so through school. Individually, the majority of students from ECU (52%) and MCM (67%) accessed their most important pre-university music instruction and activity from the private domain. Students from the UWA countered this result with 50% having accessed their most important instruction and activity through school and 44% privately. This result reflects that more UWA music students hailed from selective government funded in-school specialized music programs that operated in Western Australia (WA). ECU commencing students were more likely to originate from more diverse pre-university schooling backgrounds (34%). The state of Victoria did not operate selective government funded in-school specialized music programs. This could explain MCM's relatively low proportion of students that accessed their most important pre-university music instruction through school (22%). Two per cent of commencing ECU music undergraduates accessed their most important pre-university music instruction from some “other” source. From the qualitative inputs from this result the most important source was revealed to be “myself.” This response was curious in that it suggested that no important music instruction and activity was sourced from either private or school access points.

### Pre-university music instruction and activities—experience emphasis and focus

**Figure 5:**
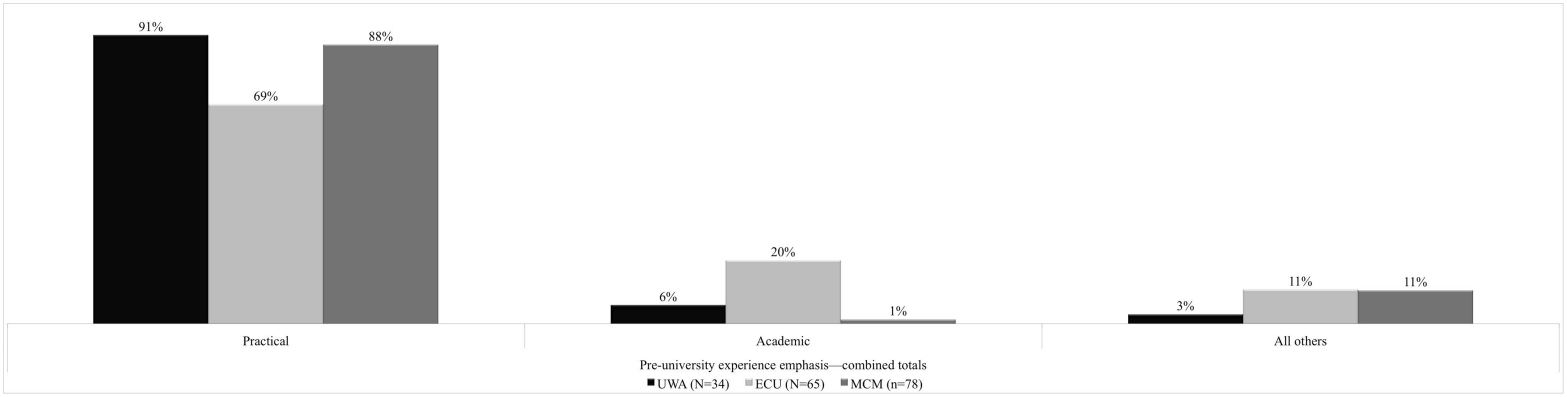
**Percentage combined pre-university music experience emphasis**.

**Figure 6:**
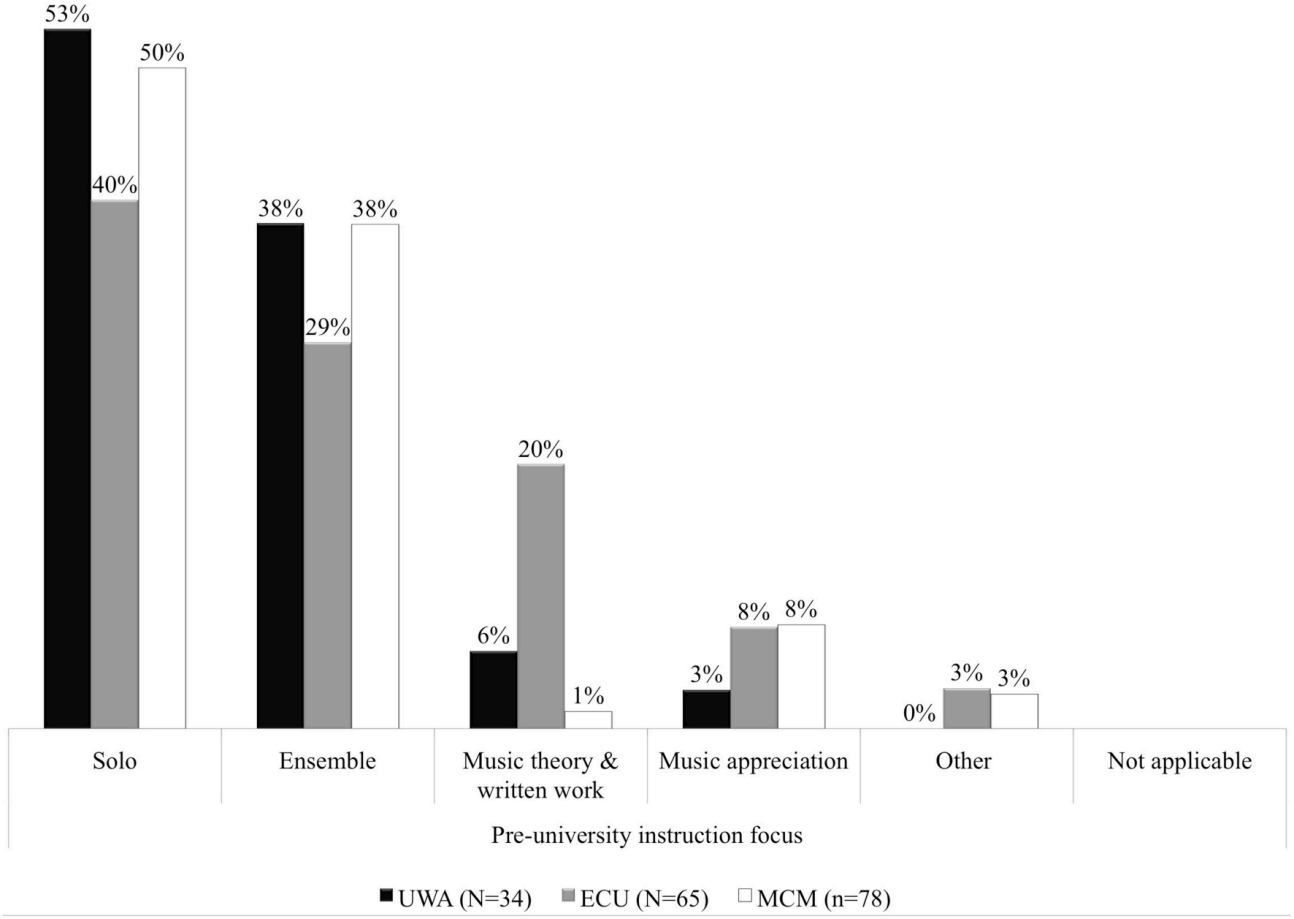
**Percentage focus of pre-university music instruction**.

The data in Figure 5 show that 83% of commencing music undergraduates received an emphasis on practical, performance-based experiences in their pre-university training. Almost 9% had an academic emphasis, and 8% had experiences that emphasized other aspects of musical encounter (addressed further in Figure 6). At the individual level in Figure 5, ECU's result for academic experience (20%) suggests that some within this cohort had musicological experiences perhaps encountered in high school through composition. The ECU and MCM results for “All others” (11% each) suggests that commencing music undergraduates were drawn from an eclectic demography. There was significantly less of an emphasis on pre-university academic experiences for those students entering UWA (6%) and MCM (1%). The majority of students had a pre-university music instruction emphasis on practical music making.

Figure 6 follows from Figure 5 to show the breakdown into the direction, or focus, that pre-university music instruction encouraged students to concentrate upon. Combining and averaging the data across the groups, of the 83% (Figure 5) that had practical based pre-university experiences, 48% of these experienced this through an individualized (solo) and 35% a participative (ensemble) instructional focus. Within these distributions, UWA and MCM were comparable. Fifty-three per cent and 50% respectively, of UWA and MCM commencing undergraduates experienced a solo focus in pre-university instruction, and 38% each through ensemble. ECU commencing undergraduate data suggests s a more graduated declination of representation across five of the six categories (see Figure 6). The spread of ECU results (from 40 to 3%) may be reflective of the diversity of background from where ECU music undergraduates originated. The academic focus (music theory and written work) remained unchanged across the three schools, Figure 5. “All other” instructional focus was predominantly experienced in the domain of music appreciation, with some data (3%) unable to be specifically defined.

### Liberalized university education—undergraduate expectations of music study

**Figure 7:**
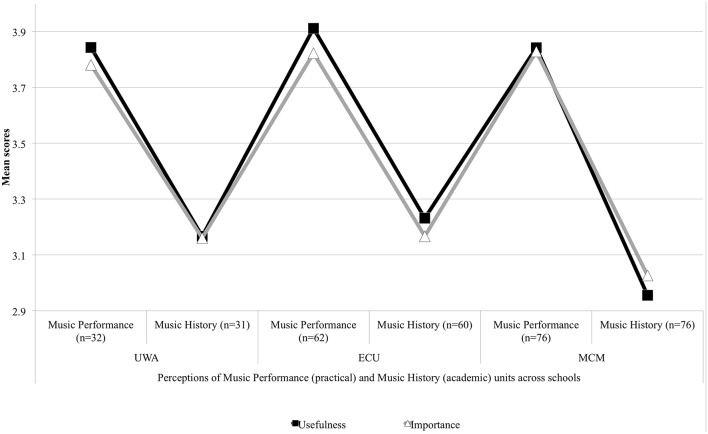
**Expectations of university practical and academic units**.

The data in Figure 7 show consistently that the commencing music undergraduates expected Music Performance (practical) units would be of greater usefulness and importance to them than Music History (academic) units. MCM undergraduates displayed the lowest expectations of the groups toward how they would perceive the usefulness (*M* = 2.96) and importance (*M* = 3.03) of the Music History unit.

**Figure 8:**
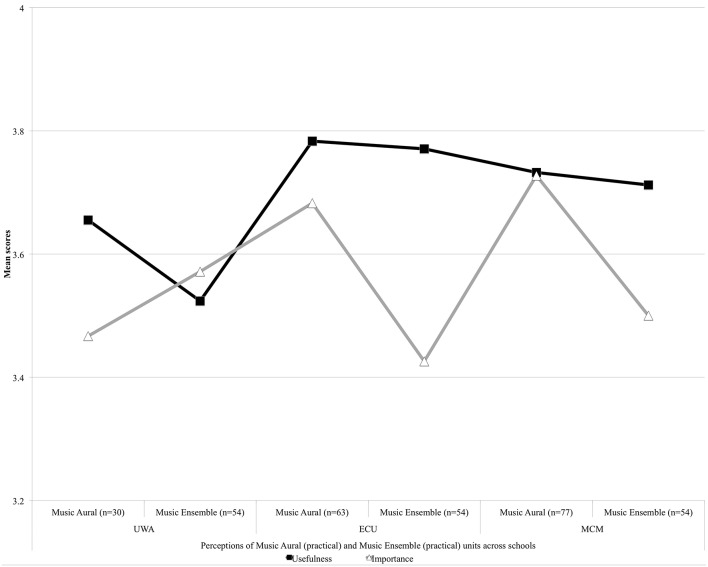
**Expectations of other university practical units**.

Undergraduate expectations toward other practical units of study generically referred to as Music Aural and Music Ensemble are reported in Figure 8. For UWA students, Music Ensemble came under the Second Major and for MCM students, under the Applied Skills unit. Overall, music undergraduates across the schools perceived that Music Aural would be useful to them. Music Aural would be slightly less important excepting for MCM students, who expected Music Aural would be equally useful and important (*M* = 3.73). ECU students would find Music Aural to be most the useful (*M* = 3.78). ECU's result for Music Ensemble's importance (*M* = 3.43) shows the greatest deviation amongst the groups from the unit's perceived usefulness (*M* = 3.77). Expectations toward the usefulness and importance of these other practical units were nonetheless positive across the groups.

**Figure 9:**
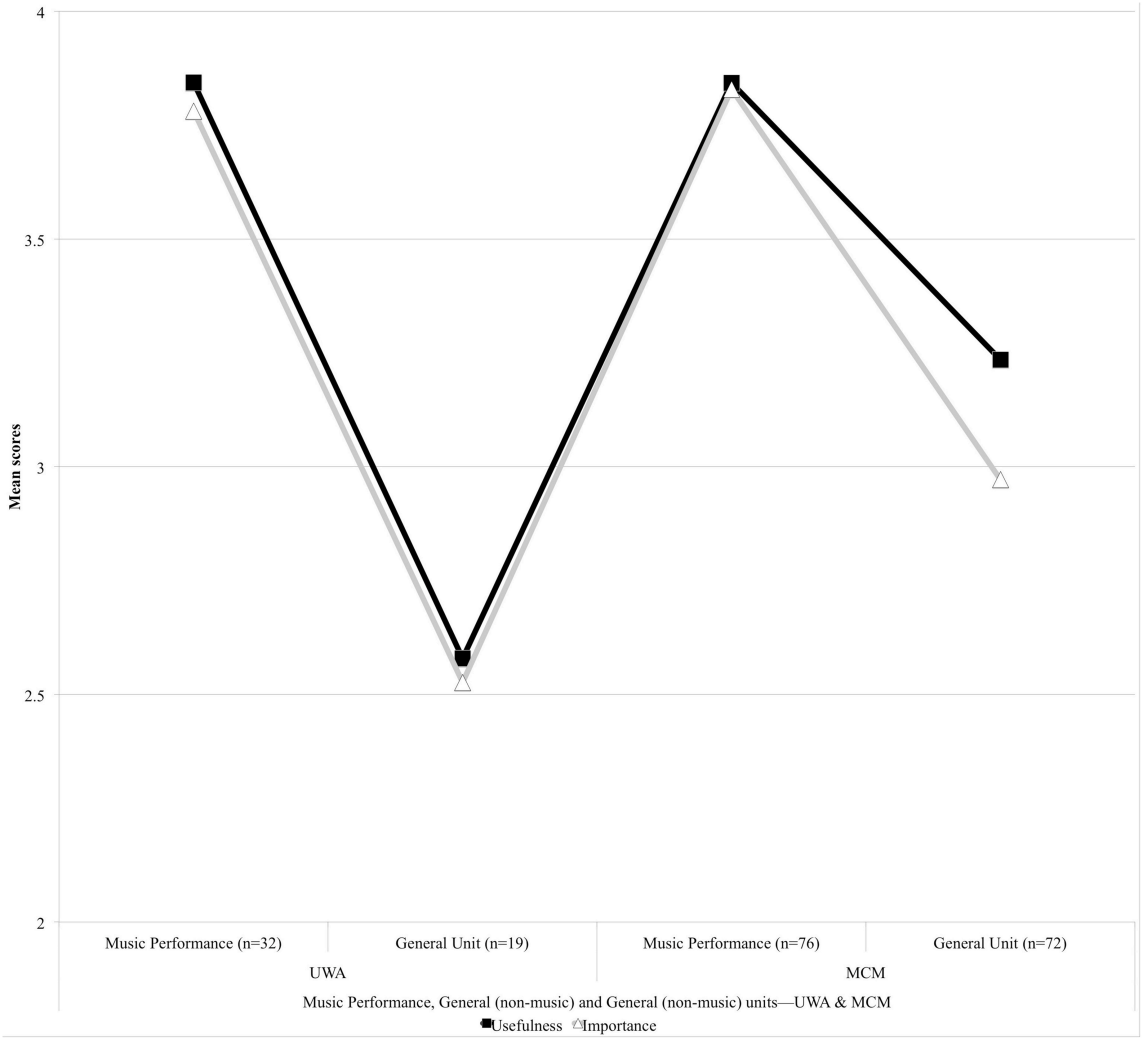
**Expectations of university worldview (General) units**.

Figure 9 shows only results for UWA and MCM, as these institutions were the ones to have implemented the liberalized worldview Broadening and Breadth (General) units. The results for Music Performance have been included here as a comparison. The UWA results showed that expectations of the Broadening Unit's usefulness (*M* = 2.58) and importance (*M* = 2.53) were significantly lower than those for Music History (see Figure 7). In contrast, MCM undergraduates (Figure 9) anticipated that the Breadth Unit would be marginally more useful (*M* = 3.24) to them than the Music History unit (*M* = 2.96, Figure 7). Conversely, the Music History unit (*M* = 2.97, Figure 7) would be almost as important to them as the Breadth Unit (*M* = 3.03, Figure 9).

Spearman's rho correlation tests were performed across the entire dataset for the three universities presented in Table 6. Quantitative results revealed strong positive relationships between usefulness and importancev expectations and for all units of music undergraduate study. With alpha set at 0.05, in all cases the results achieved significance at *p* < 0.01. Mean (*M*) scores revealed the least confidence from the UWA, ECU, and MCM cohorts toward Music History (*M* = 3.10 Usefulness, *M* = 3.10 Importance) compared to Music Performance, Music Aural and Music Ensemble. For UWA and MCM, mean scores suggest that the General unit (Broadening and Breadth) would not be as useful (*M* = 3.09) or important (*M* = 2.88) as the other units. Standard Deviation (*SD*) scores showed increased levels of disagreement that Music History would be useful and important across the cohorts for Usefulness (*SD* = 0.790) and Importance (*SD* = 0.796). The levels of disagreement were stronger still for UWA and MCM in expectations that the General unit (Broadening and Breadth) would be useful (*SD* = 0.844) or important (*SD* = 0.867) to music undergraduates. Music History and the General unit showed the greatest variance in cohort perceptions that these units would be as useful and important to music undergraduates.

**Table 6:** **Usefulness (U) and Importance (I): correlation, mean, standard deviation, variance across combined UWA, ECU, and MCM (*n* = 177)**.

**Spearman's rho**	**Correlation**	**Mean**		**Standard deviation**		**Variance**	
	U*I	**U**	**I**	**U**	**I**	**U**	**I**
Music performance (*n* = 159)	0.702^**^	3.87	3.82	0.423	0.431	0.179	0.185
Music history (*n* = 133)	0.635^**^	3.10	3.10	0.790	0.796	0.624	0.634
Music aural (*n* = 159)	0.525^**^	3.74	3.66	0.532	0.595	0.283	0.354
Music ensemble (*n* = 133)	0.648^**^	3.70	3.48	0.519	0.659	0.270	0.435
General UWA and MCM (*n* = 87)	0.753^**^	3.09	2.88	0.844	0.867	0.712	0.752

** Correlation is significant at the 0.01 level (1-tailed).

The correlations in the Table for each unit of study were between Usefulness and Importance (U*I). Mean, standard deviation and variance have been reported each for Usefulness (U) and Importance (I).

## Discussion

Expectation behaviors presented in the results aligned in large part to the literature addressing choice decisions based upon familiarity perceptions toward the known rather than the unknown (Fishbein and Ajzen, 1975; Ajzen, 1991; Hunt, 2006). That is, the results reflected more positive expectations for the practical units of study providing more useful and important experiences and benefits than theoretical/academic units. In this respect, it seems that commencing music undergraduates relied upon their experiential familiarity with pre-university music instruction and activities to inform their predictions of imminent university music study. Results also demonstrated that music undergraduate expectations were strongly informed by the way in which access to music instruction had emphasized music performance as the primary form of music activity prior to university. The first assumption that commencing music undergraduates would be positive in their expectations of university study was supported. In other words, commencing music undergraduates' positive holistic expectations of the degree program may be explained by simple enthusiasm that their first year of university study was about to begin. The second assumption that expectations would reflect specific subjective value preferences toward utility in particular areas of study also was supported and aligned also with the literature (e.g., Wigfield and Eccles, 2000; Eccles and Wigfield, 2002). For example, these value preferences reflected more positive feedback toward the expected utility of practical units like Music Performance rather than theoretical/academic units like Music History.

Importantly, the study reported an emphasis across all pre-university student experiences on an individual focus: Solo music making. This result was consistent with the literature, in that one-to-one tuition was a preferred focus for pre-university music instruction the majority having been accessed through the private domain (e.g., McPherson and Davidson, 2006; McPherson and O'Neill, 2010). In the context of the liberal arts-styled university curriculum, this result was an important one, as the tenet underlying the model is to broaden the undergraduate experience through a generalized educational framework (e.g., Reinalda and Kulesza, 2006; Bennett, 2008a). The specialized vs. liberalized debate would be worth further investigation in relation to its impact upon undergraduate music education. The solo instructional focus result did not diminish the important roles that ensemble and composition instruction played in both high school and private domains. However, the observations highlighted an area for additional considered review. For example, it was of some concern that attitudes toward the utility of theoretical/academic units such as Music History and General units (Broadening and Breadth) appeared to be negatively reinforced through the pre-university instruction and experience process. In other words, discriminatory preferences were developed through the predominant expectation that music study was primarily about performing. These attitudes toward distinguishing between the relative usefulness and importance of practical and theoretical/academic-based disciplines could only have been developed through the pre-university music education process. This finding requires further investigation, particularly as it impacts how music undergraduates are developed throughout their high school educations.

Despite the fact that it was not a focus of the study, the results generated some interesting data around the issue of daily solitary deliberate practice. Only a very small percentage of the commencing music undergraduates had committed themselves to 3 h or more of daily solitary deliberate practice prior to university (e.g., Ericsson et al., 1993; Ericsson, 2006). Although the item choice options did not discriminate exacting timed increments, the fact that the majority had not committed to 3 h or more per day would pose several interesting questions. Among these questions would be: How seriously Australian music undergraduates perceived solitary daily practice to be considering that research has supported its importance in achieving “best expert” mastery (e.g., Amirault and Branson, 2006; Ericsson, 2006; Lehmann and Gruber, 2006); how competitive Australian music undergraduates considered themselves to be in carving out a future in the world through music; what aspirations Australian music undergraduates held concerning the possibilities that university music study would help them to attain. One caveat would be appropriate at this point; as Lehmann and Gruber (2006) have argued, not all musical instruments would be capable of sustaining large amounts of sustained daily deliberate practice. Some musical instruments would be taken up at a later stage than others. For example, voice. Participant interpretation of the questionnaire item may have eliminated a holistic response. For example, “I practice my main instrument for 1 h…but I also compose music, practice my aural singing, and learn my recorder parts for the early music group I'm involved in.” The results and interpretations of these data therefore would need to be considered in such light.

A possible limitation in the study was the length of the questionnaire and the time it took to complete. This was ~25 min, though the students who picked up surveys largely completed and handed them in. The researchers considered the possibility that participants might be subject to questionnaire fatigue. As of the writing up of the current study, researchers are satisfied that for commencing undergraduates, completed questionnaires appeared to maintain integrity throughout in terms of participant responses. An issue that was factored into the study but that could not be controlled for was the type of music undergraduate participant. For example, if a student was of standard university entrance age or mature age, or majored in classical guitar as opposed to electric guitar. Such issues did not appear to affect the results, as all commencing music undergraduates from each participating school were offered the same individual degree program units of study. Researchers foresaw this latter point and the questionnaire's generic construction allowed participants flexibility to contribute information that might lie outside of the respective university handbook entry as presented in the questionnaire.

### Conclusions

There was no observed effect that the Bologna styled model of music undergraduate education had any negative influence upon UWA music undergraduate expectation perceptions. The same could be said of the other models operating at the respective participating universities. Practical university music units were perceived to be both useful and important to commencing undergraduates across the three schools studied. This in itself was an important observation as neither the liberalized nor the conservatoire based structures appeared to affect undergraduate perceptions. Practical units were expected to have greater utility than academic and general units and universally across UWA, ECU, and MCM cohorts. Previous experiences appeared to strongly influence these perceptions and were relied upon to inform music undergraduate expectations. Pre-university music training and experience emphasis mainly prepared students for music performance in individual and ensemble contexts; that academic music study was perceived less of an applied attribute to this emphasis; that this emphasis affected music undergraduate expectations in that music performance would continue as the major focus of music undergraduate study. What the study contributes is the suggestion of a possible disconnect of purpose between music training and experience prior to university and that encountered at university. This is particularly the case for the research-intensive universities (UWA and UM). This suggestion may have implications for the planning of performance education degree programs in the future. As the study involved commencing music undergraduates only, it would be of further interest to investigate how and in what ways cohort expectations toward units of study changed as the undergraduate degree program progressed. It would be also of further interest to investigate differences between female and male participants particularly regarding pre-university educational influences and their respective expectations of university music study.

## Author contributions

DH was responsible for the research design, data collection, analyses, and preparation of the manuscript. JD and CN provided interpretive, creative, and technical input to the study design, analyses and revision of the manuscript.

### Conflict of interest statement

The authors declare that the research was conducted in the absence of any commercial or financial relationships that could be construed as a potential conflict of interest.
