# Supplementary material for: Music Undergraduates' Usefulness and Importance Expectations: The Bologna Process from an Australian University Perspective
Source: Front Psychol. 2016 Jul 12;7:1054. doi: 10.3389/fpsyg.2016.01054 (PMC4940396; doi:10.3389/fpsyg.2016.01054)
Supplement: Supplementary file 1 [file DataSheet1.docx]

**Appendix**

PROJECT TITLE: ***Australian music undergraduate education: Investigating expectations, experiences, and perceptions of quality and effectiveness***

INVITATION: Welcome. You are invited to participate in research being conducted that hopes to assist Australian university music schools meaningfully inform the quality and effectiveness of music higher education in Australia.

**Researcher 1—**details withheld.

**Researcher 2—**details withheld.

**Researcher 3—**details withheld.

WHAT IS THE STUDY ABOUT? The European model of higher education, known as the Bologna Process, was implemented at the *Institution* in 2012. A similar model has been operating at other Australian universities since 2008. This research study intends to investigate the implementation of the process at *Institution* and compare this process with two other Australian university music schools. The research seeks to investigate how this introduction might affect the quality and effectiveness of music undergraduate study within these university music schools.

WHAT DOES THE STUDY INVOLVE? The study simply requires participants to respond to a questionnaire that will be distributed in a meeting forum, either through a school class or meeting.

HOW MUCH TIME WILL THE STUDY TAKE? The survey questionnaire should take participants about 20 minutes to complete.

WILL THE STUDY BENEFIT ME? The study is expected to provide information that will inform music higher education in general in Australia. The study is expected to benefit every individual within this context through enabling an opportunity to respond to questions concerning expectations, experiences and quality and effectiveness perceptions of music undergraduate education.

WILL THE STUDY INVOLVE ANY DISCOMFORT FOR ME? Apart from the time it will take to complete the survey, there is no other discomfort.

HOW IS THIS STUDY BEING FUNDED? As it forms part of the Doctor of Philosophy degree, neither competitive funding nor other commercial interests are funding this study.

WILL ANYONE ELSE KNOW THE RESULTS? HOW WILL THE RESULTS BE DISSEMINATED? All aspects of the study, including the results, will be treated in strictest confidence and in a professional manner. Only the researchers will have access to information on participants. Feedback will be freely available upon request. Any feedback will be in summary form only that will not reveal any information about individual or group participants involved in the research.

In reporting, the researcher(s) will provide an opportunity for participants to observe a final presentation on the results of the project. Participants will be debriefed on the full nature of the study and its outcomes through this presentation process. Any reporting will be made available for them to view. Participants will not be individually identifiable in the reporting of this research.

CAN I WITHDRAW FROM THE STUDY? Involvement in the study is completely voluntary. You are not obliged to be involved and if you do participate, you can withdraw at any time without giving any reason. There are no consequences or penalties should you decide to withdraw. Please sign and return the Consent to Participate form if you are happy to participate.

CAN I TELL OTHER PEOPLE ABOUT THE STUDY? Yes. You can provide anybody with the chief investigator’s contact details (name@institution.edu.au). They can then contact the chief investigator to discuss their participation in the study and obtain an information sheet.

WHAT IF I REQUIRE FURTHER INFORMATION? When you have read this information, Researcher 2 (name@institution.edu.au) will be happy to discuss it with you further and answer any questions you may have.

WHAT IF I HAVE A COMPLAINT? The *Institution* Human Ethics Research Committee has approved this study. If you have any complaints or reservations about the ethical conduct of this research, you may contact the Ethics Committee through the *Institution* Research Office. Address, <http://www.research.institution.edu.au/staff/human-research/welcome-to-HREO>. Any issues you raise will be treated in confidence and investigated fully, and you will be informed of the outcome.

**CONSENT TO PARTICIPATE**

I, (please print name)…………………………………………………………………… having read and understood the information provided, consent to participate in the research project: ***Australian music undergraduate education: Investigating expectations, experiences, and perceptions of quality and effectiveness***, being conducted by *Researcher name*.

I understand that participation in this study is completely voluntary and once undertaken, that I can exercise the right to withdraw at any time. Should I withdraw, there are no consequences or penalties for making that choice. I also understand that information provided by me will be used only in strictest confidence and only by the researchers involved directly with the study.

Signed: ____________________________________________________________

February 2013

If you would like to receive updates relating to the progress of the research, please feel free to leave us with your email address below.

1. On average, before university I practiced each day for (please check only one box [✓]):

| Less than 1 hour |  |  |  |
| --- | --- | --- | --- |
| Between 1 hour and 3 hours |  |  |  |
| Between 3 hours and 5 hours |  |  |  |
| Between 5 hours and 8 hours |  |  |  |
| More than 8 hours |  |  |  |

1. On average, before university I studied music (including reading about, writing about, history, theory, composing, aural, harmony etc.) each week for (please check only one box [✓]:

| Less than 1 hour |  |  |  |
| --- | --- | --- | --- |
| Between 1 hour and 3 hours |  |  |  |
| Between 3 hours and 5 hours |  |  |  |
| Between 5 hours and 8 hours |  |  |  |
| More than 8 hours |  |  |  |

1. The most important music instruction I received before university came from my (please check one box [✓] or check ‘Not applicable’):

| School (pre–university) music program/teacher(s) |  |
| --- | --- |
| Private (studio) music teacher(s) |  |
| Home, parents, relatives |  |
| Community music organisation |  |
| Religious group/organisation |  |
| Friends outside of school and organisations |  |
| Current tertiary instrumental teacher |  |
| Current tertiary ensemble teacher |  |
| Current tertiary academic lecturer |  |
| Other (briefly state here)________________________________________________ |  |
| Not applicable – (only check this option box if none of the above are appropriate to you) |  |

1. The most important music experiences I received focused on (please check only one box [✓] or ‘Not applicable’):

| Performing as a soloist, competing, achieving grades etc |  |
| --- | --- |
| Performing in groups like bands, choirs, orchestras, shows, etc |  |
| Musicianship for theory, aural training, composition, history etc |  |
| Music appreciation through drama and music, dance and music, listening etc |  |
| Other (briefly state here)________________________________________________ |  |
| Not applicable – (only check this option box if none of the above are appropriate to you) |  |

1. As incoming students to university Music, there are generic subjects that you will study. Depending upon your degree, some are core and central to Music study. The following asks you to rate how useful and important you think the subjects will be to you overall for this first year (Semesters 1 & 2). Please check [✓] a number from both sides of the subject choices below, or check 5, or check 6 for each subject.

| USEFULNESS | | | | | | | | | | | | | | IMPORTANCE | | | | | | | | | | | | | | | | |
| --- | --- | --- | --- | --- | --- | --- | --- | --- | --- | --- | --- | --- | --- | --- | --- | --- | --- | --- | --- | --- | --- | --- | --- | --- | --- | --- | --- | --- | --- | --- |
| Not at all useful | | Somewhat useful | | Useful | | | Very useful | | Unable to comment | | N/A unit not taken | | UNIT | | Not at all important | Somewhat important | | | Important | | | Very Important | | | Unable to comment | | N/A unit not taken | | |  |
| 1 | 2 | | 3 | | | 4 | | 5 | | 6 | | Music Performance Unit Semester 1 | | | 1 | 2 | | | | | 3 | 4 | | | 5 | | 6 | | |  |
| 1 | 2 | | 3 | | | 4 | | 5 | | 6 | | Music Aural Unit Semester 1 | | | 1 | 2 | | | | | 3 | 4 | | | 5 | | 6 | | |  |
| 1 | 2 | | 3 | | | | 4 | 5 | | 6 | | Music History Unit Semester 1 | | | 1 | | 2 | | | 3 | | | 4 | | | 5 | | | 6 | |
| 1 | 2 | | 3 | | | | 4 | 5 | | 6 | | Music Ensemble Unit Semester 1 | | | 1 | | 2 | | | 3 | | | 4 | | | 5 | | | 6 | |
| 1 | 2 | | 3 | | | | 4 | 5 | | 6 | | General Unit Semester 1 | | | 1 | | 2 | | | 3 | | | 4 | | | 5 | | | 6 | |
| 1 | 2 | | 3 | | | | 4 | 5 | | 6 | | Other Unit not listed Semester 1 | | | 1 | | 2 | | | 3 | | | 4 | | | 5 | | | 6 | |
| 1 | 2 | | 3 | | | 4 | | 5 | | 6 | | Music Performance Unit Semester 2 | | | 1 | | | 2 | 3 | | | | | 4 | 5 | | | 6 | |  |
| 1 | 2 | | 3 | | | 4 | | 5 | | 6 | | Music Aural Unit Semester 2 | | | 1 | | | 2 | 3 | | | | | 4 | 5 | | | 6 | |  |
| 1 | 2 | | 3 | | 4 | | | 5 | | 6 | | Music History Unit Semester 2 | | | 1 | | 2 | | | 3 | | | 4 | | | 5 | | | 6 | |
| 1 | 2 | | 3 | | 4 | | | 5 | | 6 | | Music Ensemble Unit Semester 2 | | | 1 | | 2 | | | 3 | | | 4 | | | 5 | | | 6 | |
| 1 | 2 | | 3 | | 4 | | | 5 | | 6 | | General Unit Semester 2 | | | 1 | | 2 | | | 3 | | | 4 | | | 5 | | | 6 | |
| 1 | 2 | | 3 | | 4 | | | 5 | | 6 | | Other Unit not listed Semester 2 | | | 1 | | 2 | | | 3 | | | 4 | | | 5 | | | 6 | |

***All information within this section is strictly confidential. It will be used only for purposes of this research.***

**Where applicable please check the appropriate box (✓)**

1. Your university Student ID number (do NOT print your name)____________________________________
2. 🞎 Male 🞎 Female

| 1. Age | 🞎 17-19 yrs | 🞎 20-24 yrs | 🞎 25-27 yrs | 🞎 28-40 yrs | 🞎 41-49 yrs | 🞎 50+ yrs |
| --- | --- | --- | --- | --- | --- | --- |

1. Your instrument (voice, sacbut, composition, musicology etc)_____________________________
2. The course you are enrolled in at university _______________________________________
3. Enrollment status 🞎 Full time 🞎 Part time 🞎 Other_________________________
4. Where did you complete your secondary schooling?

🞎 Australia (State)_________(school name)__________________________________________

🞎 Overseas (Country)____________(school name)____________________________________

THANK YOU,

WE APPRECIATE YOU TAKING THE TIME TO PARTICIPATE IN THIS SURVEY
